# Exposure and Health Risks Posed by Potentially Toxic Elements in Soils of Metal Fabrication Workshops in Mbarara City, Uganda

**DOI:** 10.3390/jox14010011

**Published:** 2024-01-30

**Authors:** Eunice Nuwamanya, Denis Byamugisha, Caroline K. Nakiguli, Christopher Angiro, Alice V. Khanakwa, Timothy Omara, Simon Ocakacon, Patrick Onen, Daniel Omoding, Boniface Opio, Daniel Nimusiima, Emmanuel Ntambi

**Affiliations:** 1 Department of Chemistry, Faculty of Science, Mbarara University of Science and Technology, Mbarara P.O. Box 1410, Uganda; enuwamanya15@gmail.com (E.N.); cnakiguli@must.ac.ug (C.K.N.); nimusiimadaniel2@gmail.com (D.N.); 2 Centre for Water, Environment and Development, School of Water, Energy and Environment, Cranfield University, Cranfield MK43 0AL, UK; christopher.angiro.602@cranfield.ac.uk; 3 Department of Environmental Health and Disease Prevention, Faculty of Public Health, Lira University, Lira P.O. Box 1035, Uganda; vonikhanakwaalice@ph.lirauni.ac.ug; 4 Department of Chemistry, College of Natural Sciences, Makerere University, Kampala P.O. Box 7062, Uganda; 5 Department of Civil and Environmental Engineering, College of Engineering, Design, Art and Technology, Makerere University, Kampala P.O. Box 7062, Uganda; ocakaconsimon@gmail.com; 6 Department of Chemistry, University of Kerala, Thiruvananthapuram 695581, India; patrickonen1995@gmail.com; 7 Department of Chemistry, Faculty of Science, University of Lucknow, Lucknow 226007, India; omodanzo@gmail.com; 8 Department of Science and Vocational Education, Lira University, Lira P.O. Box 1035, Uganda; bfopio@lirauni.ac.ug; 9 Department of Chemistry, Faculty of Science and Technology, Andhra University, Visakhapatnam 530003, India

**Keywords:** metal fabrication workshops, toxic metals, health risks, soil pollution, average daily doses, carcinogenic risk, hazard index

## Abstract

Metal fabrication workshops (MFWs) are common businesses in Ugandan cities, and especially those producing metallic security gates, window and door frames (burglar-proof), and balcony and staircase rails. The objective of this study was to comparatively assess the pollution levels and potential health risks of manganese (Mn), chromium (Cr), cadmium (Cd), lead (Pd) and nickel (Ni) in pooled surface soil samples from four 5-, 7-, 8-, and 10-year-old MFWs (*n* = 28) and a control site (*n* = 8) in Mbarara City, Uganda. The concentration of the potentially toxic elements (PTEs) was determined using inductively coupled plasma–optical emission spectrometry. Contamination, ecological, and human health risk assessment indices and models were used to identify any risks that the PTEs could pose to the pristine environment and humans. Our results showed that PTE pollution of soils is occuring in the MFWs than at the control site. The mean concentrations of the PTEs (mg kg^−1^) in the samples were: Mn (2012.75 ± 0.23–3377.14 ± 0.31), Cr (237.55 ± 0.29–424.93 ± 0.31), Cd (0.73 ± 0.13–1.29 ± 0.02), Pb (107.80 ± 0.23–262.01 ± 0.19), and Ni (74.85 ± 0.25–211.37 ± 0.14). These results indicate that the PTEs could plausibly derive from the fabrication activities in these workshops, which is supported by the high values of contamination factors, index of geoaccumulation, and the overall increase in pollution load indices with the number of years of operation of the MFWs. Human health risk assessment showed that there are non-carcinogenic health risks that could be experienced by children who ingest PTEs in the soils from the 7-, 8- and 10-year-old MFWs. The incremental life cancer risk assessment suggested that there are potential cancerous health effects of Cd and Ni that could be experienced in children (who ingest soils from all the four MFWs) and adults (ingesting soils from the 8- and 10-year-old MFWs). This study underscores the need to implement regulatory guidelines on the operation and location of MFWs in Uganda. Further research should be undertaken to investigate the emission of the PTEs during welding operations in the MFWs.

## 1. Introduction

Urbanization is a proxy of human development and progress [1]. In essence, urbanization in the Western world has been associated with economic growth and industrialization, both of which mutually and intrinsically reinforce each other [2,3]. In developing countries, urbanization is rather rapid, poorly planned, and starkly implemented. Coupled with industrialization, associated energy requirements, and weak regulatory structures, urbanization has accelerated environmental degradation, urban sprawl, slum development, inadequate urban infrastructures, human insecurity, regional inequalities, violence, crime, and social unrest [1,4].

In the 21st century, urbanization is one of the most significant global trends, as evidenced by large social, economic, physical, and environmental transformations. According to the available data, no less than 4.4 billion (56%) of the global population reside in cities, and this proportion is anticipated to reach 68% by the year 2050 [5], or 6 billion by 2045 [5]. If such an expected growth is unmistakable [6], and with the knowledge that more than 80% of the global gross domestic product is being generated in cities, urbanization could ably contribute to sustainable growth through increased productivity and innovation [5]. In Africa, the urban population grew to 567 million people in 2015 from 27 million in 1950, which translates into a 2000% increase [7]. This trend appears to hold true in all parts of the continent. Uganda, the focus of this study, has recently undergone subtle urbanization and industrial transformations, as evidenced by 15 municipalities (Arua, Gulu, Mbale, Jinja, Masaka, Mbarara, and Fort Portal in July 2020; Entebbe and Lira on 1 July 2022; Moroto, Nakasongola, Soroti, Kabale, and Wakiso on 1 July 2023) attaining city statuses [8,9,10]. However, the creation of these cities has resulted in urban restructuring and an increment in anthropogenic activities.

One of the noticeable establishments in African, East African, and, in particular, Ugandan cities and urban centers are metal fabrication workshops (MFWs) [11,12]. They deal in the production of construction materials such as security gates, metallic window and door frames (burglar proofs), and balcony and stare case rails [13,14]. In some parts of Uganda, such as Katwe (Kampala) and Eastern Uganda, MFWs are also involved in the machining and fabrication of industrial and agricultural machines [15,16]. The MFWs are characterized by activities (such as laser, waterjet, power-scissor or plasma-arc cutting, forging, drawing, grinding, punching, stamping, welding, vanishing, forming, brazing, galvanizing, and assembly to create the final product) [11,12,17] that can lead to the release of potentially toxic elements (PTEs) into the environment. The materials used in fabrication (e.g., wire electrode, welding rods and sheets, and filler metals) are made of alloyed steel consisting of PTEs such as manganese (Mn), cadmium (Cd), chromium (Cr), nickel (Ni), vanadium (V), copper (Cu), silicon (Si), cobalt (Co), zinc (Zn), and iron (Fe) [18]. PTEs, sometimes called heavy metals or toxic elements, are those chemical elements that possess high molecular weights and/or densities that are at least five-fold greater than that of water [19]. They are known to induce toxic effects when present in the body at higher concentrations. The toxic effects largely depend on the concentration, valence states, chemical form, routes and duration of exposure, and bio-availability, among other factors [20]. For example, the ferromagnetic metal Ni is an immunotoxic and carcinogenic agent which, depending on the ingested dose and exposure duration, may cause asthma, lung fibrosis, contact dermatitis, cardiovascular diseases, and reproductive toxicity, as well as respiratory tract cancer [21].

The release of PTEs into the environment following the activities of MFWs may contaminate top soil, as well as underground and surface water resources, through leaching and soil erosion [22,23,24]. Previous studies have shown that, in addition to PTEs, welding fumes also contain toxic substances such as carbon monoxide, carbon dioxide, phosgene, asbestos (a heat-protective component used to cover the weld to prevent abrupt cooling), hydrogen fluoride, ozone, and nitrogen oxides, all of which may pose an occupational hazard to welders [25,26]. The International Agency for Research on Cancer (IARC), in 1990, classified welding fumes as a possible human carcinogen (group 2B) [27]. An assessment of welding fumes’ potential carcinogenicity led to their classification as a group 1 carcinogen in humans because empirical studies associated increased risks of lung cancer with the inhalation of such fumes, especially in non-smokers [28,29].

Metal fabrication activities have long been ongoing in Mbarara City, Uganda, due to the city’s rapid growth [30]. To date, no study has examined the emission rate, or the effect of these activities on the contamination of the soils, nearby water sources, and the atmosphere. This study, conducted for the first time in Uganda, assessed the pollution levels and potential human and ecological health risks of PTEs (Mn, Cr, Cd, Pd, and Ni) in surface soil samples from four MFWs in Mbarara City, Western Uganda.

## 2. Methods

### 2.1. Study Area Description

**Figure 1:**
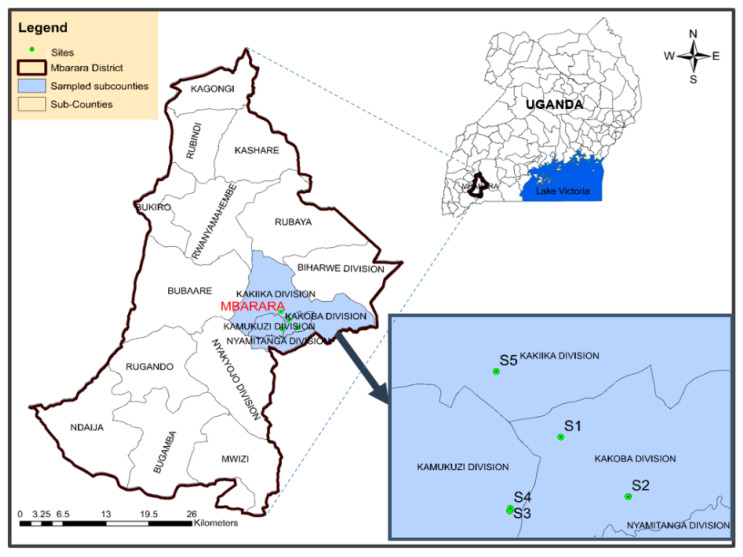
Map showing the location of the studied metal fabrication workshops in Mbarara City boroughs, Uganda. Sampling sites S1–S4 and S5 refer to the metal fabrication workshops and the control site, respectively.

Mbarara City is located in Western Uganda (latitude −0.607160 and longitude 30.654503), about 270 km from Kampala City on Kampala-Kabale Road. Its name is traced to the tall green grass “*Emburara*” (*Hyperenia ruffa* in binomial taxonomy) which covered the whole area. Its development into an urban center is traced to the appearance of Asian traders up to 1950 [31]. The city covers a total land area of 51.47 square kilometers, built on hilly areas separated by short, small, and shallow valleys. It lies at an average altitude of 1432 m above sea level [32]. It is divided into two constituencies (Mbarara City South and Mbarara City North). These are further divided into six boroughs (Biharwe, Kakiika, Kakoba, Kamukuzi, Nyakayojo, and Nyamitanga) and 23 wards. Mbarara town was granted city status in May 2019, but this only started on 1 July 2020 (Figure 1). Since then, it has remained the principal commercial center of Western Uganda. The city had an estimated population of 221,300 people as of 2020 [33].

**Figure 2:**
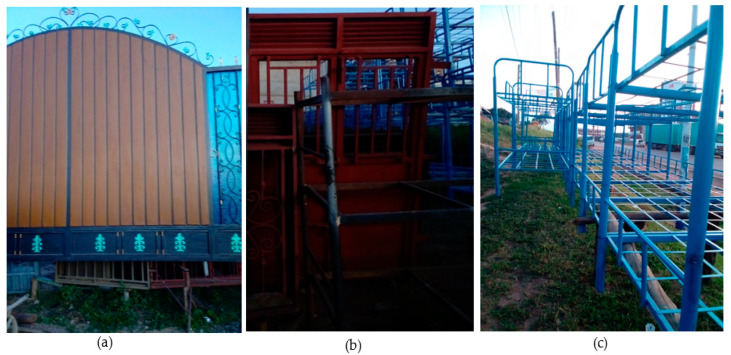
Some of the fabricated products at the metallic fabrication workshops considered in this study: (**a**) a security gate, (**b**) a metallic door and window, and (**c**) metallic double-decker beds.

In this study, four MFWs were purposely selected, and these were coded as sampling sites S1 (0°35′34.3″ S 30°40′00.2″ E) and S2 (0°36′12.3″ S 30°40′41.4″ E) in City South Division, and S3 (0°36′21.4″ S 30°39′28.9″ E) and S4 (0°36′19.8″ S 30°39′29.3″ E) in City North Division (Figure 1). They had been producing security gates, metallic doors, windows, and double-decker beds (Figure 2) for 5, 7, 8, and 10 years, respectively. A control site for this study (S5; 0°34′52.3″ S 30°39′20.6″ E) was a grassland, purposely selected in Nyarubanga village, Mbarara City North Division, about 5 km from the business area, which is comparatively less populated. Secondly, there were no MFWs that were likely to introduce PTEs into the S5 soils.

### 2.2. Sampling Procedure

**Figure 3:**
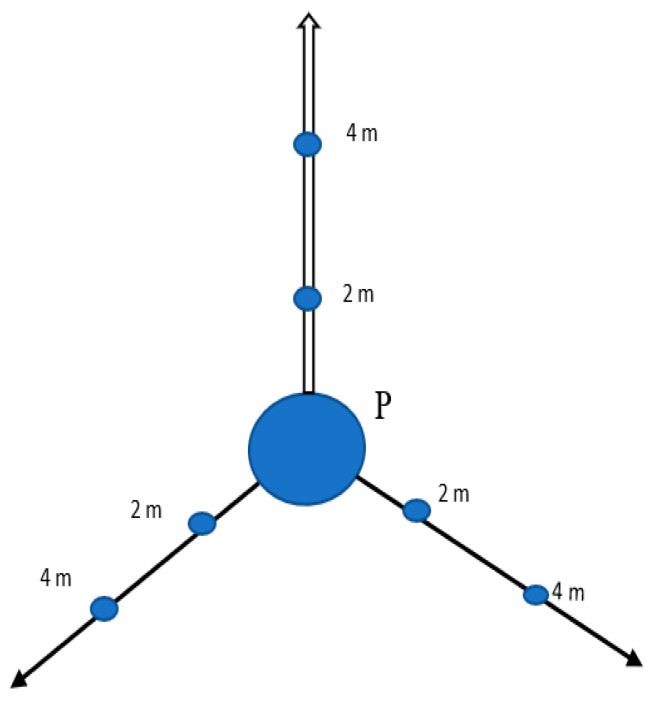
Approach used when sampling soils from metal fabrication workshops in Mbarara City, Uganda.

Soil samples were collected over a period of one month (November 2021) from each of the selected MFWs using systematic–judgmental sampling technique. From each workshop, the soil (about 0.5 kg) was collected using a clean, depth-calibrated soil auger at a depth of 0–30 cm [34]. The auger was cleaned using tissue paper and 70% ethanol after each sample collection. From each workshop, soil samples were collected as follows: (i) the point at which the metal rods and sheets are cut (this constituted the central sampling point (P), (ii) 2 m away from P, and (iii) 4 m away from P. This was carried out in three different directions for each workshop from P, as illustrated in Figure 3.

The soil samples from each workshop were pooled into an analytical sample, mixed, and then packed into labelled clean polythene bags. They were sealed, packed into a cool, dry ice box, and transported to the Chemistry Laboratory of Mbarara University of Science and Technology, Mbarara, Uganda. The soil samples were air-dried for 14 days, ground and sieved through a 0.5 mm sieve, and packed awaiting analysis.

### 2.3. Determination of PTEs Concentration

The air-dried soil sample (1 g) was weighed into a digestion vessel. To this, 9 mL of 37% hydrochloric acid and 3 mL of 69% nitric acid were added. The vessel and its contents were put in a microwave (Multiwave 3000, Anton Paar, Graz, Austria), and the following operating conditions were employed (ramp time: 5 min; MW power: 1400 W; hold time of 10 min). The contents were cooled for 20 min. After cooling, the digest was transferred into a 100 mL volumetric flask and made up to the mark with deionized water. The soil samples were analyzed for total concentrations of PTEs (Mn, Cr, Cd, Pd, and Ni) using an inductively coupled plasma-optical emission spectrometer (ICP-OES; Optima 7000 DV, Perkin Elmer Inc., Waltham, MA, USA). The wavelengths used were 257.610, 267.716, 214.440, 220.353, and 231.604 nm, respectively.

Quality control and quality assurance activities performed in this study included the analysis of blanks and spiked samples, which provided recoveries in analytically acceptable ranges (from 98.5 to 100.5%). Relative Standard Deviations of the experiments, as a measure of the precision of the analytical method, varied from 4.2% to 4.9%. All the reagents and chemicals used were of high analytical purity, supplied by Sigma-Aldrich (St. Louis, MO, USA), HiMedia Pvt Laboratories (Mumbai, India), or Merck (Darmstadt, Germany), and were used in the analysis as supplied, without further purification.

### 2.4. Human Exposure and Health Risk Assessment

Health risk assessments were performed to examine the relationship between the environment and human health. The risks were categorized into carcinogenic and non-cancer risks, for both children and adults. The chronic daily intake (CDI) (mg kg^−1^day^−1^) of the PTEs through incidental ingestion (CDI_ING_), inhalation (CDI_INH_), and dermal contact (CDI_DC_) was calculated using Equations (1)–(3) [35]. The factors used are described in the Supplementary Materials (Table S1) [35,36,37,38,39].

(1) $${\mathrm{CDI}}_{\mathrm{ING}}=\frac{C_{PTE}\times IngR\times CVF\times FI\times EXF\times EXD}{W_{ab}\times T_{aet}}$$

(2) $${\mathrm{CDI}}_{\mathrm{INH}}=\frac{C_{PTE}\times InhR\times EXF\times EXD}{{PEF\times W}_{ab}\times T_{aet}}$$

(3) $${\mathrm{CDI}}_{\mathrm{DC}}=\frac{C_{PTE}\times S_{AF}\times CVF\times AF\times DAF\times EXF\times EXD}{W_{ab}\times T_{aet}}$$

#### 2.4.1. Non-Carcinogenic Health Risk Assessment

The hazard quotient (HQ) was calculated to establish non-cancer risks from the PTEs through the three different exposure routes (Equations (4)–(6)). Due to the known additive effects of PTEs, the hazard index (HI) was established as the sum of the HQ of the individual element through a given exposure pathway (Equation (7)) [40].

(4) $${\mathrm{HQ}}_{\mathrm{ING}}=\frac{{CDI}_{ING}}{{R_{f}D}_{ING}}$$

(5) $${\mathrm{HQ}}_{\mathrm{INH}}=\frac{{CDI}_{INH}}{{R_{f}D}_{INH}}$$

(6) $${\mathrm{HQ}}_{\mathrm{DC}}=\frac{{CDI}_{DC}}{{R_{f}D}_{DC}}$$

(7) $$\mathrm{HI}=\sum_{i=1}^{n=5}HQ$$

where R*_f_*D_ING,_ R*_f_*D_INH_, and R*_f_*D_DC_ are the oral (direct ingestion), inhalation, and dermal reference doses of the specific element. The R*_f_*D_ING_ = 0.0460, 0.001, 0.01, 0.0035, and 0.02 mg kg^−1^ for Mn, Cr, Cd, Pb, and Ni, respectively. The corresponding values for R*_f_*D_INH_ and R*_f_*D_DC_ are 0.0000143, 0.0000286, 0.001, 0.000525, and 0.00540 mg kg^−1^day^−1^ and 0.0460, 0.000006, 0.001, 0.035, and 0.0206 mg kg^−1^day^−1^ [35,36,37,38,40].

#### 2.4.2. Carcinogenic Health Risk Assessment

The cancer risk (CR), estimated as the incremental lifetime cancer risk for ingestion of the carcinogenic elements (Cd, Pb and Ni), was calculated using Equation (8) [35].

CR = CDI × CSF(8)

where CSF is the ingestion cancer slope factor = 5.01 ×10^−1^, 8.5 ×10^−3^ and 8.4 × 10^−1^ mg kg^−1^day^−1^, respectively, for Cd, Pb, and Ni [35].

### 2.5. Assessment of Soil Pollution Levels

To establish the pollution levels of the soil samples from the MFWs, we computed the geoaccumulation indices, as follows: contamination factor, geo-accumulation index, pollution load index, and potential ecological risk index. They were calculated with respect to the concentrations of PTEs in the control site (S5). Firstly, the contamination factor was calculated using Equation (9), suggested by Hakanson [41]. The index of geoaccumulation (I_geo_), used by Müller [42] in the first instance, was computed (Equation (10)).

(9) $$\mathrm{Contamination}\;\mathrm{factor}\;({\mathrm{CF}}_{s})=\frac{C_{PTES}}{C_{BKG}}$$

(10) $$I_{\mathrm{geo}}={\mathrm{Log}}_{2}\;\frac{C_{PTES}}{{1.5\;C}_{BKG}}$$

where C_PTES_ = element concentration in the soil sample, C*_BKG_* = background concentration of the same element in soil from S5, and 1.5 is the background matrix correction factor [43,44]. The classification of these indices (CF_s_ and I_geo_) is described in Table S2. To discern the cumulative pollution load in a particular soil sample, the pollution load index (PLDI) was calculated using Equation (11) [40].

PLDI = (CF_S1_ × CF_S2_ × CF_S3_ × CF_S4_ × CF_S5_)^1/5^ (11)

where CF_S1_ to CF_S5_ are the calculated contamination factors for the five elements analyzed in this study. Lastly, the potential ecological risk index technique based on the work of Hakanson [41] was used to assess the sensitivity of the biotic community to the PTEs. The potential ecological risk coefficient ($E_{R}^{i}$) and potential ecological risk index (PERI) were calculated (Equations (12) and (13)).

(12) $$E_{R}^{i}=T_{R}^{i}\times {\mathrm{CF}}_{s}$$

(13) $$\mathrm{PERI}=\sum_{i=1}^{i=n}E_{R}^{i}$$

In these equations, $T_{R}^{i}$ = biological toxic factor of the PTEs: Cd = 30, Pb = Ni = 5, Cr = 2 and Mn = 1 [45]. The risk characterization criterion is provided in Table S2.

### 2.6. Statistical Analysis

The quantitative data were analyzed using GraphPad Prism for Windows (version 9, GraphPad Software, San Diego, CA, USA). Descriptive statistics were summarized as means with standard deviation for the measured PTEs concentration. The data were normalized, and one-way analysis of variance (ANOVA) was used to establish the significant differences in the means within and between groups, using Tukey multiple comparison test. Pearson’s bivariate correlation analysis was used to establish the nature of the interrelationships between the concentration of PTEs in the MFWs, as well as the years in which the workshops had been in existence. The relationship was considered significant at *p* ≤ 0.05 if not *p* ≤ 0.01.

## 3. Results

### 3.1. Concentration of the PTEs in the Soil Samples

**Figure 4:**
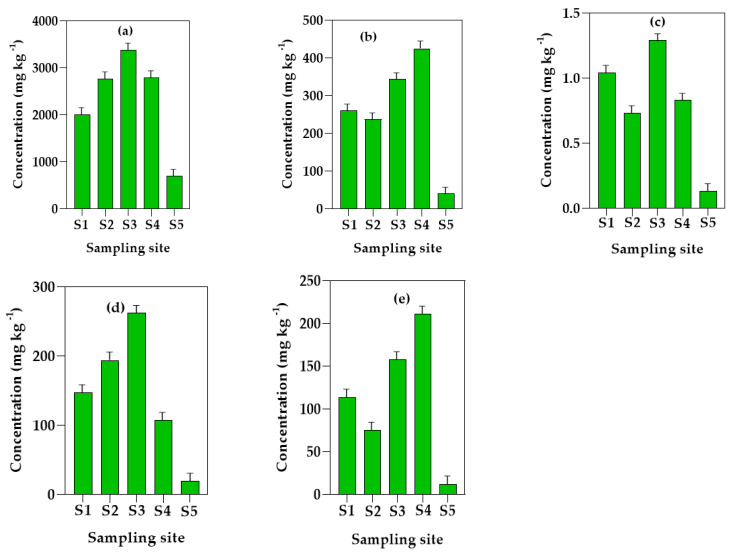
Concentration of PTEs in soils from MFWs in Mbarara City, Uganda: (**a**) manganese, (**b**) chromium, (**c**) cadmium, (**d**) lead, and (**e**) nickel. The sampling sites S1–S4 and S5 refers to the MFWs and the control site, respectively. The method detection limits for the PTEs were 0.03, 0.25, 0.07, 1.4, and 0.4 mg kg^−1^ for Mn, Cr, Cd, Pb, and Ni, respectively.

The results for the PTEs concentration obtained in the soil samples from the four MFWs and the control site are shown in Figure 4. On average, the mean concentrations of the PTEs followed the order Mn > Cr > Pb > Ni > Cd. These differences between the mean values of the individual PTEs in the MFWs were, however, not statistically significant according to the one-way ANOVA results (*p* > 0.05). The concentration of Mn ranged from 2012.75 ± 0.23 mg kg^−1^ at S2 to 3377.14 ± 0.31 mg kg^−1^ at S4. The concentrations of Mn in the soils from the four MFWs were higher than that of the control site, S5 (695.30 ± 0.004 mg kg^−1^). Chromium had the second highest concentration among the PTEs in the soil samples. The levels ranged from 237.55 ± 0.29 mg kg^−1^ at S1 to 424.93 ± 0.31 mg kg^−1^ at S3. Significant amounts of cadmium were also found in the soils from all the sampling sites but in lower quantities than the rest of the metals. Its total concentration varied from 0.73 ± 0.13 mg kg^−1^ at S1 to 1.29 ± 0.02 mg kg^−1^ at S4. Lead was also found in considerable amounts, ranging from 107.80 ± 0.23 mg kg^−1^ at S3 to 262.01 ± 0.19 mg kg^−1^ at S4. On the other hand, the concentrations of Ni that were recorded ranged from 74.85 ± 0.25 mg kg^−1^ at S1 to 211.37 ± 0. 14 mg/kg at S3. In all cases, the values were higher than the corresponding concentrations obtained at the control site (S5).

### 3.2. Inter-Elemental Correlation of the Concentration of the PTEs in the Soil Samples

**Figure 5:**
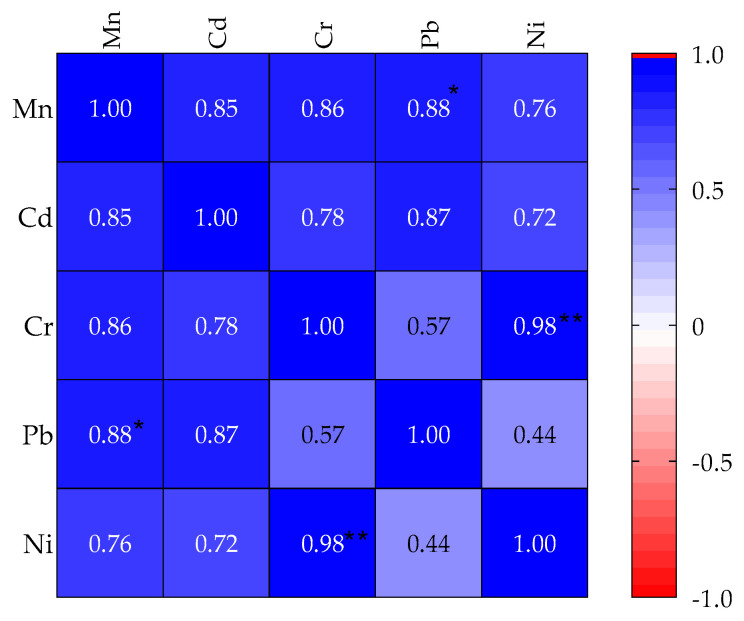
Pearson’s correlation coefficient (*r*) matrix plot for the interrelationships between the PTEs of the soils from the sampled MFWs in Mbarara City, Uganda. * Significant at the 0.05 level (2-tailed); ** also significant at 0.01 level (2-tailed).

The results of Pearson’s pairwise correlation (*r*) for the PTEs concentration in the soil samples from the four MFWs and the control site are shown in Figure 5. On average, there were weak to strong positive correlations between the PTEs (*r* = 0.44 to 0.98). However, Ni and Cr (*r* = 0.98, *p* = 0.0039) and Mn and Pb (*r* = 0.88, *p* = 0.051) pairs had strong and significant positive correlations.

### 3.3. Human Health Risk Assessment Indices

#### 3.3.1. Non-Cancer Risk Results

In this study, the chronic daily intake through incidental ingestion (CDI_ING_) ranged from 9.3 × 10^−6^ mg kg^−1^ day^−1^ for Cd at S1 by children to 5397.2 × 10^−6^ mg kg^−1^ day^−1^ for Mn ingested by adults at S4 (Table 1). These values were, however, higher than the calculated values for soils sampled from the control site (S5). The hazard indices ranged from 0.0833 at S2 for adults to 1.7531 at S4 for children. With the exception of S1 and S5, there are potential non-cancer health effects that could arise from the ingestion of soil from the MFWs by children.

On the other hand, the calculated CDI_INH_ ranged from 7.7 × 10^−15^ mg kg^−1^ day^−1^ for Cd at S1 by children to 32,810.1 × 10^−15^ mg kg^−1^ day^−1^ for Mn ingested by children at S4 (Table 2). The corresponding average hazard quotients (0.25 × 10^−10^ to 22,944.13 × 10^−10^) and hazard indices (1877.67 × 10^−10^ to 22,945.70 × 10^−10^) for average chronic intake through inhalation (Table 2) never surpassed 1, suggesting that no adverse health effects would likely be experienced by individuals who inhale the PTEs in soils from the MFWs. 

The values for CDI_DC_ ranged from 0.026 × 10^−6^ mg kg^−1^ day^−1^ for Cr at S1 by children to 120.90 × 10^−6^ mg kg^−1^ day^−1^ for Mn at S4. For adults, the intakes ranged from 0.046 × 10^−6^ mg kg^−1^ day^−1^ for Cr at S1 to 215.35 × 10^−6^ mg kg^−1^ day^−1^ for Mn at S4 (Table 3). None of the computed hazard quotients and hazard indices for the intake of the PTEs in the soils through dermal absorption surpassed 1, attesting to the absence of any discernable health effects for both groups. It is also worth noting that, in all cases, the calculated chronic doses, hazard quotients and indices for the control site (S5) were lower than those of the samples from the MFWs.

#### 3.3.2. Cancer Risk Estimations

The incremental life cancer risk assessment indicated that the risk values ranged from 1.2 × 10^−6^ for Pb at S3 to 272.2 × 10^−6^ for Cd at S4 (Table 4). Thus, there are potential cancer risks in children who ingest soils from all the four MFWs, as the cancer risk values for Cd and Ni (at S2–S4) and the total cancer risk values were outside the acceptable range of 1 × 10^−6^ to 1 × 10^−4^ indicated by the US EPA. In this case, Cd and Ni are the major drivers of carcinogenic health effects in children.

For adults, cancer risk values ranged from 0.10 × 10^−6^ for Pb at S3 to 141.9 × 10^−6^ for Ni at S3. In adults, the individual cancer risk values for Ni and the total cancer risk values at S3 and S4 lay outside the safe limit of 1 × 10^−6^ to 1 × 10^−4^, alluding to potential carcinogenic health effects. Interestingly, Ni was the sole driver of carcinogenic health effects in adults.

### 3.4. Pollution Level and Ecological Risk Assessment Indices

**Figure 6:**
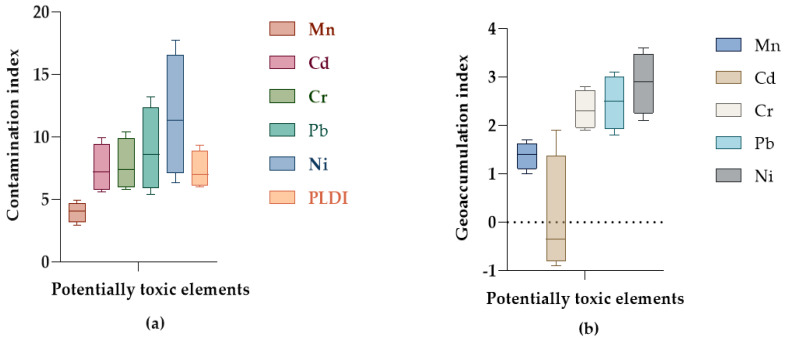
Box-and-whisker plot of (**a**) contamination factors and pollution load index (PLDI), and (**b**) geoaccumulation indices of the PTEs in soils from some metal fabrication workshops in Mbarara City, Uganda.

The effect of fabrication activities on soil pollution was assessed. The results of CFs, I_geo_ and pollution load indices for the MFWs are shown in Figure 6. The results of CF_s_ calculation showed that the values in the classification ranged from 1 ≤ CF_s_ ≤ 3 and 3 ≤ CF_s_ < 6 for Mn, and 3 ≤ CF_s_ < 6 to CF_s_ > 6, for all the other PTEs (Figure 6a). For the PLDI (Figure 6a), the calculated values spanned from 6.0 for soils from S1 to 9.3 for soils from S4. Taken together, PTE pollution of the soils tended to increase with the years of operation. The level of pollution showed a positive correlation with the period for which the MFWs had been in existence (*r* = 0.76, *p* < 0.05).

On the other hand, the indices of geoaccumulation (I_geo_) calculated for the soil samples from the MFWs spanned from −0.9 for Cd at S3 to 3.6 for Ni at S3 (Figure 6b). In this index, the PTEs had the following order: Ni > Pb > Cr > Mn > Cd. An assessment of $E_{R}^{i}$ and PERI due to PTEs contamination of the soils obtained values ranging from 2.9 for Mn at S2 to 297.0 for Cd at S4, and 264.1 at S1 to 400.2 at S4, respectively (Table 5). The risk, in order of severity, followed the chemical sequence: Cd > Ni > Pb > Cr > Mn.

## 4. Discussion

### 4.1. Concentration and Inter-Elemental Corrlation of the PTEs in the Soil Samples

Uganda does not have its own guideline limits for PTEs in soils. The World Health Organization (WHO) target values indicate that the desirable maximum levels of PTEs in unpolluted soils are 0.8, 100, 85, and 35 mg kg^−1^ for Cd, Cr, Pb, and Ni, respectively [46]. All the PTEs exceeded their respective target values in the MFWs, as well as the global averages of the PTEs in the upper continental crust (571.0, 0.40, 59.5, 27.0, and 29.0 mg kg^−1^ for Mn, Cd, Cr, Pb, and Ni, respectively) reported by previous studies [47,48]. These results indicate that there is PTE contamination of the MFW soils.

The levels of Mn found in MFW soils were from 2.89 to 4.88 times higher than those found in the control site (S5). Mn is known to occur at high levels in soils but the levels observed for soils from the MFWs in this study could be due to the fact that Mn contributes the highest percentage of the composition of steel alloys (11–14.5%) that are used in welding and fabrication [49]. Exposure to Mn, an essential component of welding electrodes and some steels, has been implicated in neurotoxicity and Parkinsonism of welders [50,51].

Chromium, which had the second highest concentration among the PTEs in the soil samples, was from 2.4 to 4.3 times higher than the WHO target value, and from 5.9 to 10.4 times higher than the value at the control site (S5). These high levels could also be attributed to Cr, which is present in welding rods. It is now established that Cr, in hexavalent form (Cr^6+^), is released during welding operations [52,53,54]. Moreover, welders inhaling Cr^6+^ are at an increased risk of lung, nasal, sinus, and stomach cancers [55].

Cadmium, another constituent of welding materials, was 0.9 to 1.6 times higher than the WHO target value, and 5.6 to 9.9 times higher than the concentration obtained from the control site (S5). Cadmium is usually plated with substrate materials, such as prefabricated steel, various types of iron, and industrial fasteners, to act as a sacrificial coating [56]. It is a preferred plating material due to its beneficial properties, such as its high resistance to saltwater corrosion, outstanding conductivity, and low electrical resistance during welding. It also provides a low friction surface. When Cd-plated materials are exposed to the atmosphere, Cd corrodes and breaks down before the underlying material. In addition, some Cd may be released into the atmosphere or deposited on the ground during welding, cutting, or grinding [57]. Exposure to welding fumes has been implicated in increased urinary Cd and β2-microglobulinaemia, which implies that it leads to renal tubular dysfunction [58].

Lead, a well-known probable carcinogen, was 1.3 to 3.1 times higher than the WHO target value, and 5.4 to 13.2 times higher than the value at the control site (S5). Lead is added to steel bars and sheets to improve their mechanical properties and corrosion resistance. When exposed to air or water, Pb oxides and carbonates are formed, which protect the underlying metal from further attack. The alloying of medium carbon steel with Pb improves the cutting speeds, grinding, or filling of the steel before welding, and makes it easy to cut steel to the desired shape and smoothen joints [59]. When added, Pb does not mix completely with the metal, since it is slightly soluble in liquid or solid steel. Lead remains separate from the rest of the metal components and mechanically disperses in the steel as submicroscopic metallic inclusions when it solidifies [59]. Through cutting, grinding, filling, and welding, particulates and fumes of Pb can be generated, which end up in the soil. In addition to being a constituent of steel, Pb contamination could also occur as a result of using Pb-based paints such as red oxide to spray finished welded materials. Long-term exposure to Pb is known to induce Pb poisoning and hypertension [60,61]. The World Health Organization has also indicated that at least 900,000 deaths per year, and up to 30% of the global burden of developmental intellectual disability, are due to Pb poisoning [62]. Furthermore, inorganic Pb compounds are probable human carcinogens [63].

Finally, Ni occurred at concentrations ranging from 2.1 to 6.0 times higher than the WHO target value, and 6.3 to 17.7 times higher than the value at the control site (S5). Nickel is a metal used in the plating of steel due to its ability to increase hardening, improve the toughness and ductility of the steel, and greatly enhance resistance to atmospheric corrosion. Also, steel alloys with nickel corrode to a certain extent, after which no further corrosion takes place. Additionally, Ni-based steel is easy to form and weld. All these properties make Ni of great importance in the manufacture of steel. Occupational exposure to Ni in welding has been reported [25], and such exposure has been associated with genotoxicity, carcinogenicity, and immunotoxicity [64].

There is a paucity of published literature on PTEs in soils from MFWs. In a welding workshop in Lagos (Nigeria), Adu et al. [22] reported the presence of Cd (0.248 mg kg^−1^), Cr (8.6 mg kg^−1^), Ni (0.96 mg kg^−1^), Pb (2.469 mg kg^−1^), Zn (46.875 mg kg^−1^), and Cu (1.292 mg kg^−1^) in soils sampled from the area. Although the levels of Cd obtained in their study are comparable to the results of the current study, the other PTEs quantified in this study are higher than those reported in the Nigerian welding workshop. Another, similar study of soils sampled at 0–10 cm in welding workshops at Old Panteka Market, Kaduna (Nigeria), found the following PTEs: Cr (0.001–161.73 mg kg^−1^), Cu (15.68–3446.43 mg kg^−1^), Cd (1.28–11.23 mg kg^−1^), Pb (95.85–1220.45 mg kg^−1^), and Ni (9.35–170.78 mg kg^−1^) [23]. The observed differences in the PTE levels in MFW soils in Mbarara City when compared with previous studies could be due to differences in the depth of the sampled soils, the sampling strategies used, the number of years the workshops had been in operation prior to soil sampling, the volume of anthropogenic activities, the physicochemical properties of the soils, and the climatic conditions in the different locations [65]. 

Considering the positive correlations between the studied PTEs in the soil samples, the significant correlation coefficients between pairs of PTEs are indicative of their origination from a common source, i.e., MFWs’ activities [66,67,68], mutual dependences, or identical behaviors during transport in the environment. Elevated concentrations of siderophilic elements (Ni, Cr, Mn, and Pb) are markers of anthropogenic pollution, supporting the idea that they could have occurred as a result of the metallurgical activities in the MFWs [69].

### 4.2. Human Health Risks Posed

Pertaining to non-cancer risks, this study established that, with the exception of S1 and S5, there are potential non-cancer health effects that could arise from the ingestion of soils from the MFWs by children. This high level of exposure through ingestion in children could be because of their high hand-to-mouth habit of ingestion of things they come into contact with [35,70]. As expected, exposure to the PTEs through inhalation and dermal contact had hazard quotients that never exceeded 1. These results suggested that no adverse health effects would likely be experienced by individuals who inhale or are exposed through dermal contact with PTEs in the soils from the MFWs. This is supported by the fact that, for such exposures to occur, the soils would have to be inhaled/adsorbed as dust; therefore, these may not be the most common routes of exposure to the PTEs. A previous study in welding workshops from Potiskum town (Nigeria) showed that the highest individual hazard quotient due to ingestion of contaminated soils was contributed by Cr (6.581 × 10^−2^), but all the hazard quotients, as well as the hazard indices, never exceeded 1, indicating no potential non-cancer health risks [24].

The carcinogenic risk assessment results indicated that cancer risks may be experienced in children who ingest soils from all the four MFWs. Cd and Ni were identified as the driving carcinogenic PTEs in children, and therefore this risk could be exacerbated where children’s playgrounds are situated in the vicinity of the MFWs [35]. For adults (representing the general population), cancer risk values ranged from 0.10 × 10^−6^ for Pb at S3 to 141.9 × 10^−6^ for Ni at S3. In adults, cancer health effects were likely to be mostly driven by Ni. This showed that individuals working in the aged MFWs may be at a higher risk of developing certain cancers. These results are in agreement with a study in Nigeria [24], which concluded that Cr was the main driver of carcinogenic health risks due to the ingestion of PTE-contaminated soils from some welding shops in Potiskum town.

### 4.3. Soil Pollution Levels and Ecological Risks Posed

The effect of fabrication activities on soil pollution was assessed. The results of the CF assessment showed moderate to considerable pollution with respect to Mn, and moderate to very high pollution by all the other PTEs according to Hakanson’s classification (Table S2) [41]. Our results were comparable to a study by Jimoh et al. [23], where, for CFs, the highest CFs were observed for Pb in the soils of a welding workshop and the lowest in Cr. For the PLDI, all the soils were categorized as polluted (PLDI > 1) by the PTEs, as per the pollution grades of Tomlinson et al. [71]. The positive correlation between the PTE pollution of the soils and the number of years of operation of the MFWs indicates that there is enrichment of the PTEs in the soils. Nevertheless, the volume of the fabricated materials produced by the MFWs could also be another factor contributing to the increased levels of pollution observed.

On the other hand, the I_geo_ calculations indicated that the MFWs were practically uncontaminated with respect to Cd, except for S1, which showed median contamination levels. All the sites showed median contamination (for Mn) and from median to serious contamination for the other PTEs. A similar study in welding workshops in Nigeria found that I_geo_ ranged from −0.63 to 1.58 at 0–5 cm and −0.63 to 1.71 at 5–10 cm, respectively, indicating uncontaminated to slightly contaminated soils [23].

An assessment of $E_{R}^{i}$ and PERI revealed that there are considerable ecological risks posed by the PTEs in the MFW soils, specifically with respect to Cd, Ni, and Pb (Table 5). These results attest to the fact that the biodiversity of the studied sites is at low to high ecological risks.

## 5. Conclusions

The results of this study showed that all the MFWs had variable concentrations of PTEs, which surpassed their WHO target values in unpolluted soils and their levels in the control site by 0.9 to 17.7 times. Therefore, it is probable that the fabrication activities taking place within the MFWs are potential sources of the PTEs in the soils, which puts public health at a risk. Direct ingestion was the main exposure pathway associated with significant health-risk tendencies for soils in the 7-, 8-, and 10-year-old MFWs. The pollution indices (CFs, I_geo_, and PLDI) indicated that the soils in the MFWs are considerably to extremely polluted. This indicates a high likelihood that the polluted soils may end up in water sources, thereby compromising water quality. There is, therefore, an urgent need for the development of clear and well-planned policies pertaining to the setting-up of MFWs in Mbarara City and Uganda as a whole. Further studies should investigate the emissions of PTEs, as well as urinary or toenail biomarkers, in the welders of the MFWs.

## Figures and Tables

**Table 1:** Chronic daily intake through the incidental ingestion of PTEs in soils from MFWs in Mbarara City, Uganda.

Age Group	Sampling Site	Average Chronic Daily Intake (×10^−6^ mg kg^−1^ day^−1^)					Hazard Quotient					Hazard Index ^1^
		Mn	Cr	Cd	Pb	Ni	Mn	Cr	Cd	Pb	Ni	
Children	S1	3531.7	9.3	303.7	247.9	95.7	0.076	0.093	0.304	0.071	0.0047	0.5487
	S2	25,724.2	13.3	333.1	188.7	144.7	0.559	0.133	0.333	0.054	0.0072	**1.0862**
	S3	35,786.3	10.6	424.9	137.8	270.2	0.778	0.106	0.425	0.039	0.0135	**1.3615**
	S4	43,178.1	16.5	543.3	335.0	201.3	0.939	0.165	0.543	0.096	0.0101	**1.7531**
	S5	889.0	1.7	52.02	25.42	15.2	0.019	0.017	0.052	0.007	0.0008	0.0958
Adults	S1	4414.7	1.17	24.68	20.14	59.81	0.096	0.0012	0.0025	0.0058	0.0030	0.1085
	S2	3216.7	1.67	27.07	15.33	90.45	0.070	0.0017	0.0027	0.0044	0.0045	0.0833
	S3	4472.3	1.33	44.14	11.20	168.90	0.097	0.0013	0.0044	0.0032	0.0084	0.1143
	S4	5397.2	2.06	35.68	209.36	125.81	0.012	0.0021	0.0036	0.0060	0.0063	0.0300
	S5	1111.2	0.21	4.23	15.89	9.52	0.0024	0.00021	0.0004	0.00045	0.00048	0.0039

^1^ Values in **bold** exceeded 1, indicating the possibility of non-cancer health effects being experienced by the exposed group.

**Table 2:** Average hazard quotients and indices for chronic intake through the inhalation of PTEs in soils from MFWs in Mbarara City, Uganda.

Age Group	Sampling Site	Average Chronic Daily Intake (×10^−15^ mg kg^−1^ day^−1^)					Hazard Quotient (×10^−10^)					Hazard Index (×10^−10^)
		Mn	Cr	Cd	Pb	Ni	Mn	Cr	Cd	Pb	Ni	
Children	S1	2683.7	7.07	230.78	188.37	72.72	1876.70	0.25	0.23	0.36	0.13	1877.67
	S2	19,547.3	10.1	253.12	143.39	109.95	13,699.40	0.35	0.25	0.27	0.24	13,700.50
	S3	27,193.2	8.05	322.87	109.34	205.32	19,016.20	0.30	0.32	0.21	0.38	19,017.40
	S4	32,810.1	12.54	412.84	254.56	152.96	22,944.13	0.44	0.41	0.48	0.28	22,945.70
	S5	675.5	1.29	39.53	19.32	11.55	472.38	0.05	0.004	0.04	0.02	472.49
Adults	S1	3354.64	0.89	18.75	15.3	45.45	2345.90	0.03	0.19	0.29	0.08	2346.49
	S2	2444.3	1.27	20.57	11.65	68.73	1709.30	0.04	0.21	0.22	0.12	1709.89
	S3	3398.4	1.01	33.54	8.51	128.34	2376.50	0.04	0.34	0.16	0.24	2377.28
	S4	4101.22	1.57	27.11	15.91	95.6	2867.99	0.05	0.27	0.30	0.18	2868.79
	S5	844.38	0.16	3.21	12.07	7.23	590.48	0.06	0.03	0.23	0.01	590.81

Note: Soils from S1–S4 and S5 are soil samples taken from the MFWs and the control site, respectively.

**Table 3:** Chronic daily intake through dermal contact, hazard quotients, and indices for chronic intake through inhalation of PTEs in soils from MFWs in Mbarara City, Uganda.

Age Group	Sampling Site	Average Chronic Daily Intake (×10^−6^ mg kg^−1^ day^−1^)					Hazard Quotient					Hazard Index
		Mn	Cr	Cd	Pb	Ni	Mn	Cr	Cd	Pb	Ni	
Children	S1	98.89	0.026	0.85	0.69	0.27	0.0029	0.0043	0.00085	0.000019	0.000013	0.008082
	S2	72.06	0.037	0.93	0.53	0.41	0.0016	0.0062	0.00093	0.000015	0.000020	0.008565
	S3	100.20	0.029	1.52	0.38	0.76	0.0022	0.0048	0.00152	0.000011	0.000037	0.008568
	S4	120.90	0.046	1.23	0.94	0.56	0.0026	0.0077	0.00123	0.000027	0.000027	0.011584
	S5	24.89	0.005	0.15	0.07	0.04	0.0005	0.0008	0.00015	0.000002	0.000014	0.001466
Adults	S1	176.15	0.046	7.57	6.18	2.39	0.0038	0.0077	0.00757	0.00018	0.000116	0.019410
	S2	128.35	0.066	8.31	4.71	3.61	0.0028	0.0110	0.00831	0.00013	0.000175	0.022415
	S3	178.48	0.052	13.55	3.44	6.74	0.0039	0.0087	0.01355	0.00010	0.000327	0.026493
	S4	215.35	0.082	10.95	8.35	5.02	0.0047	0.0137	0.00110	0.00024	0.000243	0.019983
	S5	44.34	0.008	1.29	0.63	0.38	0.0010	0.0013	0.00129	0.00002	0.000018	0.003628

Note: Soils from S1–S4 and S5 are soil samples taken from the MFWs and the control site, respectively.

**Table 4:** Cancer risks through incidental ingestion of PTEs in soils from metal fabrication workshops in Mbarara City, Uganda.

Age Group	Sampling Site	Cancer Risk (×10^−6^)			Total Cancer Risk (×10^−6^)
		Cd	Pb	Ni	
Children	S1	**152.2**	2.1	80.4	**234.7**
	S2	**166.9**	1.6	**121.5**	**290.0**
	S3	**212.9**	1.2	**227.0**	**441.1**
	S4	**272.2**	2.8	**169.1**	**444.1**
	S5	26.1	0.2	12.8	39.1
Adults	S1	12.4	0.17	50.2	62.77
	S2	13.6	0.13	76.0	89.73
	S3	22.1	0.10	**141.9**	**164.1**
	S4	17.9	1.78	**105.7**	**125.38**
	S5	2.1	0.14	8.0	10.24

Values in **bold** lie outside the US EPA acceptable range of 1 × 10^−6^ to 1 × 10^−4^.

**Table 5:** Ecological risks of PTEs in soils from the metal fabrication workshops in Mbarara City, Uganda.

Sampling Site	$E_{R}^{i}$					PERI	Pollution Level
	Mn	Cd	Cr	Pb	Ni		
S1	4.0	168.0	11.6	49.0	31.5	264.1	Considerable
S2	2.9	240.0	12.8	37.0	47.5	340.2	Considerable
S3	4.1	192.0	20.8	27.0	88.5	332.4	Considerable
S4	4.9	297.0	16.8	15.5	66.0	400.2	High

## Data Availability

Data supporting the conclusions of this study are available on request from the authors.

## Supplementary Materials

The following supporting information can be downloaded at: https://www.mdpi.com/article/10.3390/jox14010011/s1, Table S1: Values of exposure factors used in health-risk assessments due to PTEs in soils from MFWs in Mbarara City, Uganda; Table S2: Classification values of pollution and risk indices used in study of PTEs in soils from MFWs in Mbarara City, Uganda.
