# Supplementary material for: Exposure and Health Risks Posed by Potentially Toxic Elements in Soils of Metal Fabrication Workshops in Mbarara City, Uganda
Source: J Xenobiot. 2024 Jan 30;14(1):176–92. doi: 10.3390/jox14010011 (PMC10885048; doi:10.3390/jox14010011)
Supplement: Supplementary file 1 [file jox-14-00011-s001.zip › jox-2750397-supplementary.pdf]

Journal of *Xenobiotics*

*Article*

# Exposure and Health Risks Posed by Potentially Toxic Elements in Soils of Metal Fabrication Workshops in Mbarara City, Uganda

**Eunice Nuwamanya [1], Denis Byamugisha [1], Caroline K. Nakiguli [1], Christopher Angiro [2], Alice V. Khanakwa [3], Timothy Omara [4,*], Simon Ocakacon [5], Patrick Onen [6], Daniel Omoding [7], Boniface Opio [8,9], Daniel Nimusiima [1] and Emmanuel Ntambi [1,*]**

1 Department of Chemistry, Faculty of Science, Mbarara University of Science and Technology, Mbarara P.O. Box 1410, Uganda; enuwamanya15@gmail.com (E.N.); cnakiguli@must.ac.ug (C.K.N.); nimusiimadaniel2@gmail.com (D.N.)
2 Centre for Water, Environment and Development, School of Water, Energy and Environment, Cranfield University, Cranfield MK43 0AL, UK; christopher.angiro.602@cranfield.ac.uk
3 Department of Environmental Health and Disease Prevention, Faculty of Public Health, Lira University, Lira P.O. Box 1035, Uganda; vonikhanakwaalice@ph.lirauni.ac.ug
4 Department of Chemistry, College of Natural Sciences, Makerere University, Kampala P.O. Box 7062, Uganda
5 Department of Civil and Environmental Engineering, College of Engineering, Design, Art and Technology, Makerere University, Kampala P.O. Box 7062, Uganda; ocakaconsimon@gmail.com
6 Department of Chemistry, University of Kerala, Thiruvananthapuram 695581, India; patrickonen1995@gmail.com
7 Department of Chemistry, Faculty of Science, University of Lucknow, Lucknow 226007, India; omodanzo@gmail.com
8 Department of Science and Vocational Education, Lira University, Lira P.O. Box 1035, Uganda; bfopio@lirauni.ac.ug
9 Department of Chemistry, Faculty of Science and Technology, Andhra University, Visakhapatnam 530003, India
* Correspondence: prof.timo2018@gmail.com (T.O.); emmantambi@must.ac.ug (E.N.)

# SUPPLEMENTARY MATERIALS

**Table S1**: Values of exposure factors used in health risk assessments due to PTEs in soils from MFWs in Mbarara City, Uganda

| Factor | Description (units) | Factor values | |
|---|---|---|---|
| | | **Children** | **Adults** |
| $C_{PTE}$ | Concentration of PTEs in the soils (mg kg⁻¹) | This study | This study |
| *IngR* | Ingestion rate (mg day⁻¹) | 200 | 100 |
| EXF | Exposure frequency (days year⁻¹) | 350 | 350 |
| EXD | Exposure duration (years) | 6 | 30 |
| FI | Fraction ingested from the contaminated source (unitless) | 1.0 | 1.0 |
| *InhR* | Inhalation rate of soil as dust ($m^3$ day⁻¹) | 200 | 100 |
| CVF | Conversion factor (kg mg⁻¹) | $1 \times 10^{-6}$ | $1 \times 10^{-6}$ |
| $W_{ab}$ | Body weight (kg) | 15 | 60 |
| $T_{aet}$ | Average time (days) | Non-carcinogenic: EXD × 365 days year⁻¹; carcinogenic: 60 years × 365 days year⁻¹ | |
| $S_{AF}$ | Surface area of skin exposed to soil as dust ($cm^2$) | 2800 | 5700 |
| PEF | Particular emission rate ($m^3$ kg⁻¹) | $1.316 \times 10^9$ | $1.316 \times 10^9$ |
| AF | Skin adherence factor (mg cm⁻²) | 0.2 | 0.7 |
| DAF | Dermal absorption factor (unitless) | 0.001 | 0.001 |

Based on US EPA [36,37], Ferreira-Baptista and De Miguel [38], and Ajeh et al. [35].

**Table S2:** Classification values of pollution and risk indices used in the study of PTEs in soils from MFWs in Mbarara City, Uganda

| Pollution and risk indicators | Classification values | Description |
|---|---|---|
| Contamination factor ($CF_S$) [41] | CF< 1 | Low contamination |
| | 1 ≤CF< 3 | Moderate contamination |
| | 3 ≤ CF< 6 | Considerable contamination |
| | CF> 6 | Very high contamination |
| Geo-accumulation index ($I_{geo}$) [42] | $I_{geo}$<0 (class 0) | Practically uncontaminated |
| | 0<$I_{geo}$<1 (class 1) | Low to median contamination |
| | 1<$I_{geo}$<2 (class 2) | Median contamination |
| | 2<$I_{geo}$ <3 (class 3) | Median to strong contamination |
| | 3<$I_{geo}$<4 (class 4) | Serious contamination |
| | 4<$I_{geo}$< 5 (class 5) | Serious to extreme contamination |
| | $I_{geo}$ > 5 (class 6) | Extreme contamination |
| Pollution load index (PLDI) | PLDI < 1 | Unpolluted |
| | PLDI > 1 | Polluted |
| Ecological risk ($E_R^i$ and PERI) [41] | $E_R^i$ < 40; PERI > 95 | Low contamination |
| | 40 ≤ $E_R^i$ ≤ 80; 95 ≤ PERI ≤ 190 | Moderate contamination |
| | 80 ≤ $E_R^i$ ≤ 160; 190 ≤ PERI ≤ 380 | Considerable contamination |
| | 160 ≤ $E_R^i$ ≤; 320; PERI ≥ 380 | High contamination |
| | 320 ≤ $E_R^i$ | Very high contamination |
